# Total Parathyroidectomy with Subcutaneous Parathyroid Forearm Autotransplantation in the Treatment of Secondary Hyperparathyroidism: A Single-Center Experience

**DOI:** 10.1155/2018/6065720

**Published:** 2018-07-09

**Authors:** Claudio Casella, Alessandro Galani, Luigi Totaro, Silvia Ministrini, Silvia Lai, Mira Dimko, Nazario Portolani

**Affiliations:** ^1^Department of Molecular and Translational Medicine, Surgical Clinic, University of Brescia, Brescia, Italy; ^2^Department of Clinical and Experimental Sciences, Surgical Clinic, University of Brescia, Brescia, Italy; ^3^Department of Clinical Medicine, Sapienza University of Rome, Rome, Italy; ^4^Nephrology and Dialysis Unit, ASST Carlo Poma, Mantova, Italy

## Abstract

**Introduction:**

Secondary hyperparathyroidism is common in chronic kidney disease. Parathyroidectomy is indicated in refractory hyperparathyroidism when medical treatments and so the parathyroid hormone levels cannot be lowered to acceptable values without causing significant hyperphosphatemia or hypercalcemia. The aim of this study is to compare the efficacy and safety of total parathyroidectomy with subcutaneous forearm autotransplantation with total parathyroidectomy with intramuscular forearm autotransplantation.

**Materials and Methods:**

A single-center retrospective cohort study of total parathyroidectomy with forearm autotransplantation from January 2002 to February 2013 was performed. According to the surgical technique, patients were divided into an intramuscular group (Group 1) and a subcutaneous group (Group 2). 38 patients with secondary hyperparathyroidism were enrolled; 23 patients were subjected to total parathyroidectomy with parathyroid tissue replanting in the subcutaneous forearm of the upper nondominant limb, while 15 patients were subjected to replanting in the intramuscular seat.

**Results:**

A total of 38 patients (56 ± 13 years) were enrolled. In both groups, the preoperative iPTH value was markedly high, 1750 ± 619 pg/ml in the intramuscular autotransplantation group and 1527 ± 451 pg/ml in the subcutaneous autotransplantation group (*p* = 0.079). Transient hypoparathyroidism was shown in 7 patients, and 1 patient showed persistent hypoparathyroidism (*p* = 0.387). 2 patients showed persistent hyperparathyroidism (*p* = 0.816), and in 2 others was found recurrent hyperparathyroidism (*p* = 0.816); 3 of them underwent autograftectomy. The anterior compartment of the forearm nondominant limb was sacrificed in 1 case of intramuscular autotransplantation with functional arm deficit.

**Conclusions:**

The efficacy and safety of parathyroid tissue autotransplantation in the subcutaneous forearm of the upper nondominant limb is confirmed with a good rate of tissue engraftment and with a comparable number of postsurgical transient and persistent hypoparathyroidism and hyperparathyroidism incidence in both techniques. Furthermore, this technique preserves arm functionality in the case of autograftectomy. Consequently, it is our opinion that total parathyroidectomy with subcutaneous forearm autotransplantation is currently the best choice.

## 1. Introduction

Secondary hyperparathyroidism (SHPT) is common in chronic kidney disease (CKD) particularly in dialysis patients, and it is responsible for mineral bone disorders (MBD) and cardiovascular diseases [1, 2]. SHPT is characterized by an increase in the parathyroid hormone (PTH) synthesis and secretion and progressive parathyroid gland hyperplasia. There is an increase in the prevalence of SPTH (intact PTH (iPTH) > 65 pg/ml) related to the decline of eGFR levels. SHPT is present in approximately 12% of those with eGFR values > 80 ml/min/1.73 m^2^, 17% of those with an eGFR of 70–79 ml/min/1.73 m^2^, 21% of those with an eGFR between 60 and 69 ml/min/1.73 m^2^, and 56% of those with an eGFR < 60 ml/min/1.73 m^2^ [2]. The improvement of medical treatment and haemodialysis regimen in patients with CKD resulted in the decrease of severe renal hyperparathyroidism (rHPT) requiring parathyroid surgery [3, 4]. There is general agreement in suggesting parathyroidectomy in patients with CKD stages 3–5 D with severe SHPT who fail to respond to pharmacological therapy or when the therapy results in unacceptable rises in levels of serum calcium and/or phosphorus (as occurs frequently using calcitriol or vitamin D analogues) and also when medical management is not tolerated because of adverse side effects. There are three surgical approaches for parathyroidectomy: subtotal parathyroidectomy (sPTX), total parathyroidectomy (tPTX), and total parathyroidectomy with parathyroid tissue autotransplantation (AT) in the sternocleidomastoid muscle or the intramuscular or subcutaneous forearm of the nondominant limb. A consensus on the best operative management is lacking, and currently, there is no general agreement regarding the best therapeutic approach. The efficacy and safety of different surgical techniques are unclear, because the studies conducted to compare the replanting methods were performed on limited patient samples and reaching nonunivocal conclusions. Total parathyroidectomy (tPTX) with autotransplant performed by an experienced surgeon effectively reduces the levels of PTH, calcium, and phosphorus and also maintains a parathyroid tissue necessary for the proper functioning of the mineral metabolism [5–19]. The recurrence of hyperparathyroidism at the site of implantation is a serious complication, which occurs with a frequency between 7 and 9%. Autograftectomy, often needed in the case of SHPT recurrence, when performed in an intramuscular site of the forearm, often imposes the sacrifice of muscle tissue resulting in functional damage [3, 14, 15, 20, 21].

## 2. Aim of The Study

The aim of this study is to compare the efficacy and safety of tPTX with subcutaneous forearm AT with tPTX with intramuscular forearm AT and to evaluate the long-term follow-up of patients in the two surgical techniques.

## 3. Materials and Methods

### 3.1. Study Design and Subjects

We performed a retrospective cohort study on 38 patients, from January 2002 to February 2013, at the General Surgery of the Hospital of Brescia with SHPT in haemodialysis regimen submitted to total parathyroidectomy (tPTX) with parathyroid tissue autotransplantation (AT) in the intramuscular (Group 1) or subcutaneous (Group 2) site of the upper nondominant forearm.

### 3.2. Inclusion Criteria

Inclusion criteria include patients aged >18 years and <75 years with SHPT with surgery indication.

### 3.3. Exclusion Criteria

Exclusion criteria include patients over 75 years of age, patients with severe comorbidity with contraindicated surgery or ASA risk score over 3, primary or tertiary hyperparathyroidism, parathyroid carcinoma, parathyromatosis, and mediastinal parathyroid gland localization.

### 3.4. Surgery Indications

According to guidelines and experts' opinions, our surgical indications were as follows: severe renal HPT refractory to medical treatment (e.g., iPTH > 800 pg/ml, hypercalcemia, and hyperphosphatemia), uncontrollable renal secondary hyperparathyroidism on cinacalcet, intolerance to medical therapy due to adverse effects, expected long-term survival with severe symptomatic renal HPT including pruritus, intractable bone pain, advanced osteopaenia/osteoporosis, calcinosis and calciphylaxis, severe osteitis fibrosa or high bone turnover, erythropoietin-resistant anemia, and dilated cardiomyopathy [3, 6–8, 10].

### 3.5. Preoperative and Postoperative Management and Surgical Technique

All patients were investigated preoperatively by neck ultrasound colour Doppler imaging (neck US/CD) and 99mTc-methoxyisobutylisonitrile (MIBI) scintigraphy [22] of the neck and mediastinum. Total parathyroidectomy (tPTX) includes careful identification and resection of all four parathyroid glands with bilateral cervical thymectomy to remove any supernumerary glands and parathyroid nests. A 70% intraoperative drop from the baseline levels after parathyroid gland tissue removal was considerate appropriate during parathyroidectomy [23]. All parathyroids were measured and weighed before their reimplantation assessing their viability and integrity. For autotransplantation (AT), the most normal-appearing gland should be minced into 10–20 1 mm^3^ pieces [5, 6, 10, 23]. Twenty-three patients were submitted to tPTX with parathyroid tissue AT in the subcutaneous forearm of the upper nondominant limb, while 15 patients were submitted to parathyroid tissue AT in the intramuscular forearm of the upper nondominant limb. Cryopreservation of parathyroid tissue was routinely performed in 21 patients until December 2013. Cryopreservation was not performed in the remaining 17 patients according to increasing evidence that the need for delayed autotransplantation is low (1%) and that the success rate of parathyroid autotransplantation is poor after cryopreservation [24–28]. Moreover, delayed autotransplantation was never required in our experience. Monitoring of plasma calcium levels was carried out by blood sampling every 8 hours for the first 2 days during the postoperative course. Below the threshold value of 7.8 mg/dl, an infusion of calcium gluconate was performed until stabilization of the serum calcium within normal values. Considering the criteria of reference rate for the method during postoperative follow-up, patients were classified as having hypoparathyroidism (<6 months with iPTH < 10 pg/ml), persistent postoperative hypoparathyroidism (>6 months with iPTH < 10 pg/ml), transient postsurgical hyperparathyroidism (iPTH > 70 pg/ml; < 6 months), persistent postsurgical hyperparathyroidism (iPTH > 70 pg/ml; >6 months), and recurrence postsurgical hyperparathyroidism (new onset of iPTH > 70 pg/ml during follow-up). The indication for autograftectomy was based on iPTH greater than 800 pg/ml not responding to medical therapy and hypertrophy of the implanted tissue detected by imaging methods [3, 6–8, 10, 11, 22, 23]. Patients with recurrent secondary hyperparathyroidism (rSHPT) following total parathyroidectomy and autotransplantation were assessed by locoregional US/CD and MIBI scintigraphy of the neck and mediastinum and by a modified Casanova test [29] to discriminate between the graft-bearing arm and the neck as the site of the recurrence. Patients underwent long-term follow-up and iPTH assay at 6, 12, 24, 36, and 60 months.

### 3.6. Laboratory Measurements

Serum calcium (mg/dl) and serum phosphorus (mg/dl) were measured using standard automated techniques. Parathyroid hormone (iPTH) (pg/ml) was measured using a two-site assay that measures “intact” hormones.

### 3.7. Statistical Analysis

Data management and analysis were performed using IBM® SPSS® Statistics 20 for Windows® software (IBM Corporation, New Orchard Road Armonk, New York, USA). All continuous variables were expressed as mean ± standard deviation, and categorical variables were expressed as numbers (percentage). Student's *t*-tests or Mann–Whitney *U* test was performed to determine differences between groups, as appropriate. Binomial test or chi-square test was used for the comparison of categorical data. A probability value of *p* < 0.05 was considered to be statistically significant.

## 4. Results

**Figure 1:**
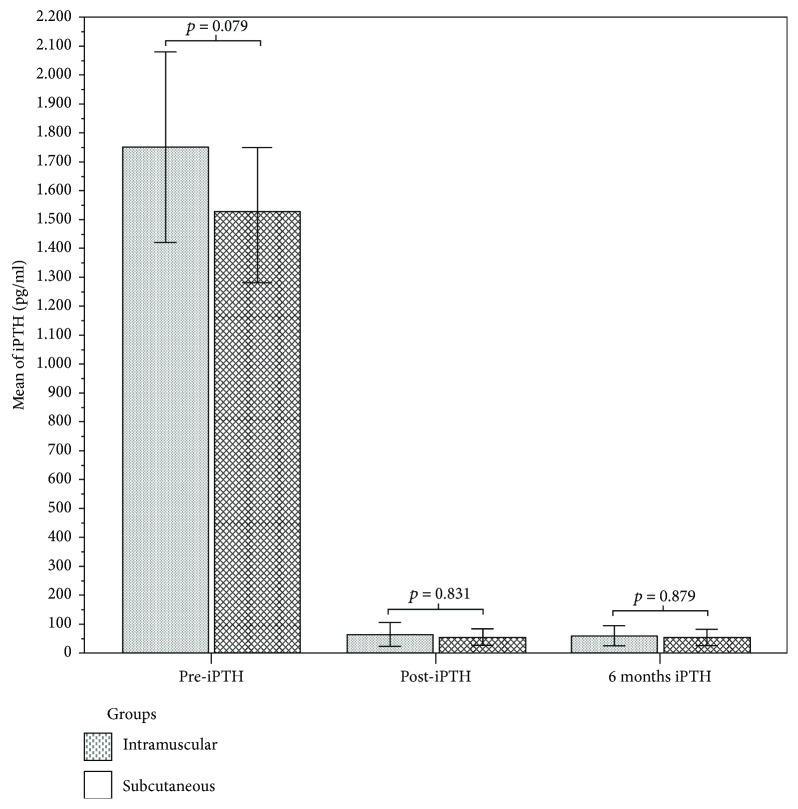
Bar charts with error bars. There were no significant statistical differences in preoperative iPTH (pre-iPTH: 1750 ± 619 pg/ml versus 1527 ± 451 pg/ml, *p* = 0.079), postoperative iPTH (post-iPTH: 63 ± 78 pg/ml versus 53 ± 66 pg/ml, *p* = 0.831), and 6 months iPTH value (6 months iPTH: 58 ± 66 pg/ml versus 53 ± 68 pg/ml, *p* = 0.879) between groups. Boxes represent means; error bars indicate standard errors.

A total of 38 patients (24 females and 12 males, mean age of 56 ± 13 years) were enrolled (Table 1). The two treatment groups were homogeneous by sex, age, duration of haemodialysis, and preoperative and postoperative iPTH values (Table 2). In both groups, the preoperative iPTH value was markedly high, 1750 ± 619 pg/ml in the intramuscular AT group and 1527 ± 451 pg/ml in the subcutaneous AT group (*p* = 0.079, Figure 1). MIBI scintigraphy of the neck and mediastinum and neck US/CD was performed in the preoperative period to identify the site of parathyroid hyperplasia. No intraoperative findings of thyroid concomitant pathology were recorded. Nobody had postoperative major complications and postsurgical bleeding, and there was no postoperative mortality. No temporary or persistent lesions of the recurrent nerve were reported. The postoperative parathyroid hormone decreased with a statistically significant difference (Figure 1, *p* < 0.001) in both study groups. Transient hypoparathyroidism was shown in 7 patients, and 1 patient showed persistent hypoparathyroidism (*p* = 0.387), both cases controlled then by medical therapy. Two patients showed transient hyperparathyroidism (*p* = 0.816), and 2 showed persistent hyperparathyroidism (*p* = 0.816). Recurrent hyperparathyroidism was found in 2 cases with nonsignificant statistic difference between the two groups (*p* = 0.816) (Table 2). Three autograftectomies were performed (*p* = 748). The anterior compartment of the forearm of the nondominant limb was sacrificed in 1 case of intramuscular AT with a functional arm deficit. The mean follow-up was 106 ± 24 months for patients undergoing intramuscular reimplantation and 103 ± 18 months for patients undergoing subcutaneous reimplantation, with no statistically significant differences (*p* = 0.710, Table 2). Moreover, the two groups of patients were homogeneous as regards the iPTH and calcium serum levels, showing a statistical homogeneity also during the follow-up.

## 5. Discussion

The need for parathyroidectomy in patients with end-stage renal disease (ESRD) is common and increases with the duration of dialysis therapy [2–5, 14]. Calciomimetic and other therapeutic agents, such as chelating phosphorus and vitamin D analogues, have been shown to be effective in secondary hyperparathyroidism, modifying the timing and necessity of parathyroidectomy in secondary and tertiary hyperparathyroidism, even if many of these drugs have a high cost to public health [3, 4, 7, 30, 31]. The poor response or specific contraindications to medical treatment direct the choice towards parathyroidectomy. Refractory hyperparathyroidism is severe, persistent, and progressive elevation of iPTH which cannot be lowered to acceptable levels with medical therapy (including vitamin D and cinacalcet analogues) without causing significant hyperphosphatemia or hypercalcemia. There is no consensus on the acceptable PTH target level that defines refractory hyperparathyroidism. We have considered iPTH > 800 pg/ml in symptomatic patients, thus, with bone degenerative involvement, hyperphosphoremia, hypercalcemia, pruritus, and osteoarticular pain. Other authors used the KDIGO guideline indicating the iPTH target threshold for treatment, a value which is nine times above the upper limit of a normal PTH assay (i.e., 585 pg/ml if the upper range of the normal assay is 65 pg/ml), even though a parathyroidectomy is generally not performed at this value. Severe hyperparathyroidism that is refractory to medical therapy and associated with hypercalcemia (in the absence of medications such as calcitriol, vitamin D, or calcium-containing phosphate binders) suggests tertiary hyperparathyroidism, in which there is autonomous secretion of PTH that is not responsive to the plasma calcium concentration [3, 5, 7, 8, 10, 11, 30–35].

The different sites for the reimplantation after parathyroidectomy both in the first intervention and in the relapses with autograftectomy are currently debated. Some studies have shown similar outcomes regarding intramuscular or subcutaneous implantation [5, 10, 12–15, 28, 36–39]. The November 2013 K/DOQI guideline confirmed that the choice of procedure may be at the discretion of the surgeons involved, and accordingly, in our center, parathyroidectomy with autotransplantation in the subcutaneous site of the upper nondominant forearm is the preferred procedure [28]. MIBI scintigraphy is considered the best test for its high sensitivity; however, its role in the localization of parathyroids before surgery is not shared and is of less impact compared to operator surgery experience, while it seems more useful in identifying the site of relapse of hyperparathyroidism [22, 40, 41]. However, in this study to improve the identification of the site of relapse and to evaluate a possible infiltration of the surrounding tissue, this technique has always been accompanied by the modified Casanova test [29] and by an ultrasound of the parathyroid implant site. As previously described in the literature, both surgical techniques appear to be effective with regard to engraftment and growth within the new anatomic site. In fact, the parathyroid tissue has high metabolic needs and also a high capacity for implantation in highly vascularized sites, such as muscle and subcutaneous tissue. The recurrence of secondary hyperparathyroidism with the need for autograftectomy is an event that occurs in 5–7.5% of cases according to the literature, but in recent studies, it was also shown to be higher than 9% with an increased risk for implantation of parathyroid tissue at the intramuscular site. According to previous studies, in the intramuscular AT, there is an increase in intraoperative time correlated with the greater difficulty for the intervention, and in the case of autograftectomy, it implies the sacrifice of an important motor component of the forearm, with consequent functional impairment and worsening of quality of life [10–15, 23, 29, 37, 40, 41]. We showed a nonsignificant difference between the two groups in the recurrence of secondary hyperparathyroidism after parathyroidectomy (only in 3 patients), while Hsu and Hung [37] showed a significant difference between the two groups; this difference could be due to the low size of the sample (7.8%).

### 5.1. Limitations

Our study presents a relatively small cohort of patients; therefore, it needs to be confirmed by further clinical studies with a larger population. It seems that future large-scale studies are needed to clarify these differences, and the prognostic relevance remains to be proven with a larger number of patients.

## 6. Conclusion

Despite the limitations of this study, the efficacy and safety of parathyroid tissue AT in the subcutaneous forearm of the upper nondominant limb is confirmed with a good rate of tissue engraftment and with a comparable number of postsurgical transient and persistent hypoparathyroidism and hyperparathyroidism incidence in both techniques. Furthermore, this technique preserves arm functionality in the case of autograftectomy. Consequently, it is our opinion that total PTX with subcutaneous forearm AT is currently the best choice.

## Figures and Tables

**Table 1:** Baseline characteristics of patients and biochemical analyses before and after total PTX with forearm AT.

	Total population
	*n* = 38
Male *n* (%)	14 (36.8)
Age (year)	56 ± 13
Dialysis vintage (months)	80 ± 44
Pre-iPTH (pg/ml)	1621 ± 532
Post-iPTH (pg/ml)	57 ± 70
<10	8 (21.1)
10–70	26 (68.4)
>70	4 (10.5)
6 months iPTH (pg/ml)	55 ± 66
<10	1 (2.6)
10–70	35 (92.1)
>70	2 (5.3)
12 months iPTH (pg/ml)	55 ± 70
24 months iPTH (pg/ml)	57 ± 72
36 months iPTH (pg/ml)	58 ± 76
60 months iPTH (pg/ml)	61 ± 93
Follow-up time after PTX + AT (months)	109 ± 19
Long-term hypoparathyroidism	1 (2.6)
Persistent hyperparathyroidism	2 (5.3)
Recurrence hyperparathyroidism	2 (5.3)
Autograftectomy	3 (7.9)

Data are show as mean ± standard deviation or number (%). Abbreviations: PTX: total parathyroidectomy; AT: autotransplantation; Pre-iPTH: preoperative intact-parathyroid hormone; Post-iPTH: postoperative intact-parathyroid hormone; 6, 12, 24, 36, and 60 months iPTH: intact-parathyroid hormone measurement at 6, 12, 24, 36, and 60 months, respectively, after PTX + AT.

**Table 2:** Comparison of group that underwent total PTX with subcutaneous forearm AT and group that underwent total PTX with subcutaneous intramuscular forearm AT.

	Intramuscular group	Subcutaneous group	*p* value
	*n* = 16	*n* = 22	
Male	6 (37.5)	8 (36.4)	*0.105*
Age (year)	51 ± 14	60 ± 12	*0.275*
Dialysis vintage (months)	89 ± 37	73 ± 47	*0.386*
Pre-iPTH (pg/ml)	1750 ± 619	1527 ± 451	*0.079*
Post-iPTH (pg/ml)	63 ± 78	53 ± 66	*0.831*
<10	3 (18.8)	5 (22.7)	*0.767*
10–70	11 (68.8)	15 (68.2)	*0.970*
>70	2 (12.2)	2 (9.1)	*0.919*
6 months iPTH (pg/ml)	58 ± 66	53 ± 68	*0.879*
<10	0 (0.0)	1 (4.5)	*0.387*
10–70	15 (93.8)	20 (90.9)	*0.748*
>70	1 (6.3)	1 (4.5)	*0.816*
12 months iPTH (pg/ml)	60 ± 71	52 ± 69	*0.819*
24 months iPTH (pg/ml)	63 ± 77	53 ± 69	*0.746*
36 months iPTH (pg/ml)	64 ± 81	54 ± 74	*0.756*
60 months iPTH (pg/ml)	66 ± 101	57 ± 89	*0.708*
Follow-up time after PTX + AT (months)	106 ± 24	103 ± 18	*0.710*
Long-term hypoparathyroidism	0 (0.0)	1 (4.5)	*0.387*
Persistent hyperparathyroidism	1 (6.3)	1 (4.5)	*0.816*
Recurrence hyperparathyroidism	1 (6.3)	1 (4.5)	*0.816*
Autograftectomy	1 (6.3)	2 (9.1)	*0.748*

Data are show as mean ± standard deviation or number (%). Abbreviations: PTX: total parathyroidectomy; AT: autotransplantation; Pre-iPTH: preoperative intact-parathyroid hormone; Post-iPTH: postoperative intact-parathyroid hormone; 6, 12, 24, 36, and 60 months iPTH: intact-parathyroid hormone measurement at 6, 12, 24, 36, and 60 months after PTX + AT.

## Data Availability

The data used to support the findings of this study are available from the corresponding author upon request.
